# Multimodal assessment of arterial stiffness using photoplethysmography and laser Doppler flowmetry

**DOI:** 10.1038/s44325-026-00115-8

**Published:** 2026-04-09

**Authors:** Parmis Karimpour, Redjan Ferizoli, James M. May, Panicos A. Kyriacou

**Affiliations:** Research Centre for Biomedical Engineering, City St George’s, University of London, London, UK

**Keywords:** Cardiology, Diseases, Health care, Medical research, Optics and photonics

## Abstract

Age-related deterioration of vascular elasticity contributes to cardiovascular disease (CVD), the leading cause of death worldwide, making early detection in primary care essential. This study investigated the feasibility of photoplethysmography (PPG) and laser Doppler flowmetry (LDF) in an in vitro vascular system, examining independent and combined use. Custom femoral vessel–tissue phantoms replicated healthy (0.82 MPa), intermediate (1.48 MPa) and unhealthy (2.06 MPa) arterial stiffness. Two blood-mimicking fluids (BMFs) with scattering agents (intralipid and LPFS) evaluated modality performance. Extracted features included area, slope ratio and datum-based PPG metrics, plus LDF DC mean flux, statistically analysed using the Kruskal–Wallis test. The intralipid-based BMF produced larger reductions in LDF DC flux. Multiclass classification improved prediction when combining signals, yielding 100% holdout accuracy for red and infra-red (IR) PPG and 95.24% for green PPG with intralipid. These findings demonstrate technical feasibility for stiffness discrimination and motivate in vivo validation against clinical markers.

## Introduction

Vascular ageing is an inevitable physiological process characterised by the gradual deterioration and loss of elasticity in blood vessels. This condition, commonly referred to as arterial stiffness, impairs vascular function by limiting the ability of vessels to expand^1^, thereby potentially disrupting normal blood flow. As vascular structures degenerate with age, the risk of developing cardiovascular disease (CVD) increases significantly. In 2021 alone, there were 648,507 recorded cases of CVD in the United Kingdom^2^, remaining its position as the leading cause of death globally. Although vascular ageing cannot be prevented, early detection and the possibility of delaying its progression are of considerable clinical interest. At present, screening for vascular ageing is not routinely available at the general practitioner (GP) level. Patients are often referred to hospital settings for vascular assessment^3^, a process that can be both daunting and time-consuming, potentially leading to delays in diagnosis. Introducing vascular ageing assessments within primary care could facilitate earlier detection, reduce patient anxiety and help optimise the use of hospital resources. Where early signs of vascular ageing are identified in GP practices, patients can then be referred for more in-depth evaluation in specialised clinical settings.

Between January 2020 and November 2023, a growing number of researchers have investigated photoplethysmography (PPG)-based techniques in preference to other commonly employed methods for assessing vascular ageing^4–8^. As such, the optical technique of PPG has been proposed as a promising approach for the development of novel vascular ageing assessment tools^9^. As arterial stiffness increases with vascular ageing, the affected vessels undergo haemodynamic, mechanical and volumetric changes. In particular, aged vessels tend to become stiffer and thicker, demonstrating reduced pulsatile expansion compared to younger, healthier vessels. The potential adoption of PPG at the primary care level is supported by increasing recognition of its broad range of applications, including oxygen saturation measurement and vital sign monitoring^10^. Furthermore, PPG is relatively low-cost, and its primary components, light-emitting diodes (LEDs) and photodiodes, can be integrated into compact, wearable devices^9,11^. PPG sensors function in two operational modes: transmission and reflectance. In transmission mode, the light source (typically an LED) is positioned opposite the photodetector, allowing light to pass through the measurement site. In contrast, the reflectance mode places both the light source and the photodetector on the same side of the measurement site, enabling the detection of light that is reflected from the tissue. When emitted, light penetrates the skin and may then be reflected, absorbed or scattered by the tissue and blood. The amount of light that reaches the photodetector is then measured^9^. As PPG is a volumetric measurement technique, its potential utility in vascular ageing assessment is based on the premise that it can detect volumetric changes associated with arterial stiffness that result from vascular ageing.

Vascular ageing is not only associated with volumetric changes but is also hypothesised to contribute to haemodynamic alterations. As arterial stiffness increases with age, it is commonly observed that pulse pressure, defined as the difference between systolic and diastolic blood pressures, increases due to a decline in diastolic pressure and a rise in systolic pressure^12^. The reduction in diastolic pressure can impair coronary perfusion, given that coronary blood flow predominantly occurs during diastole^13^. This can be explained by Poiseuille’s Law, which describes the volumetric flow rate, F, as being proportional to the pressure gradient between arterial pressure, ${P}_{a}$, and venous pressure, ${P}_{v}$, and inversely proportional to the viscous resistance, $R$, assuming laminar flow, as shown in Eq. 1. The resistance, $R$, depends on both the length of the vessel, $L,$ and the viscosity of the blood. The viscosity, µ, defined as the friction opposing fluid motion, contributes to flow resistance along with vessel geometry, as shown in Eq 2, where $r$ denotes the vessel radius^14^.

Equation 1: Poiseuille’s Law assuming laminar flow^14^.

1 $$F=\frac{{P}_{a}-\,{P}_{v}}{R}$$

Equation 2: Resistance to flow in a tube (vessel)^14^.

2 $$R=\,\frac{8{\rm{\mu }}L}{\pi {r}^{4}}$$

In cases where turbulent flow occurs, often due to pathological conditions, the relationship between pressure and flow is no longer linear. Similarly, pulsatile flow, as opposed to steady laminar flow, introduces additional resistance^15^. According to Poiseuille’s Law, stiffened, aged vessels are expected to exhibit lower flow rates due to increased resistance, in contrast to young, healthy vessels that demonstrate lower arterial stiffness and, consequently, reduced resistance. These younger vessels serve as a physiological baseline. It is important to investigate whether the progression of vascular ageing can be characterised as a deviation from this baseline, as it may be associated with altered haemodynamic patterns that impair tissue perfusion and potentially damage downstream organs^9^.

Laser Doppler Flowmetry (LDF) is a technique for assessing microvascular blood flow, with growing recognition among researchers, as evidenced by the increase in publications from 1980 to 2006^16^. The method involves illuminating the tissue surface with a laser light source, delivered via an optical fibre or a focused light beam. The probe, which integrates both the light-emitting and light-collecting fibres, captures a portion of the backscattered light. This scattered light, which results from interactions with moving red blood cells, is transmitted to a photodetector connected to signal processing electronics. The technique is based on the Doppler effect: when coherent laser light interacts with moving red blood cells, it undergoes a frequency shift (Doppler broadening). The photodetector detects this broadened frequency spectrum, and the resulting signal is processed to yield a quantitative measure of blood flow, typically displayed on a fibre optic monitor^17^. LDF has demonstrated potential as a valuable tool for the early detection of peripheral arterial disease (PAD), a condition marked by the progressive narrowing of arterial vessels, often resulting from wall thickening or the build-up of atherosclerotic plaques^9^, ^18^. Given that arterial stiffness, a consequence of vascular ageing, induces haemodynamic alterations, it is reasonable to hypothesise that changes in microvascular blood flow may also occur. This raises the question of whether vascular ageing can be indirectly monitored through such changes in blood flow, and whether LDF could serve as a viable method for its detection.

This study builds upon methodologies previously developed by the Research Centre for Biomedical Engineering, City St. George’s, University of London (RCBE)^19^, by integrating LDF into a multimodal assessment approach. The primary aim is to monitor volumetric and blood flow changes using PPG and LDF and evaluate the feasibility of this dual-modality approach for assessing vascular ageing. Three types of vessel–tissue phantoms, representing healthy, intermediate, and pathological vasculature, were fabricated at RCBE^1^ and examined within an in vitro cardiovascular system designed to replicate the environment of the lower human body. While previous research has investigated vascular health, most studies have focused on either PPG or LDF in isolation, or have relied heavily on in vivo data, which is often affected by biological variability^19–22^. In contrast, this study utilises in vitro signals from both PPG and LDF probes to explore the potential for a synergistic application of these modalities. Integration of LDF with PPG may enhance diagnostic accuracy by providing insights into volumetric changes via PPG and haemodynamic parameters via LDF. Moreover, the in vitro approach provides a controlled and repeatable platform for structured experimentation, avoiding many of the logistical and ethical constraints associated with complex in vivo studies. This multimodal approach holds promise for increasing the clinical utility of non-invasive vascular assessments, particularly in GP settings, where early identification of vascular ageing is critical for preventive cardiovascular care.

## Results

Both PPG and LDF signals were acquired from each phantom condition (healthy, intermediate, and unhealthy) for each blood-mimicking fluid (BMF), yielding a total of 12 datasets in this study. As this study was primarily an in vitro investigation, and all variables were held constant except vessel stiffness, 4-min recordings were acquired for each condition after system stabilisation, which were divided into multiple 10 s windows for the analysis. The analysis aimed to determine the technical feasibility of PPG and LDF in vitro; for in vivo translation, repeat measurements are recommended.

A custom Python script (version 3.12.3) developed by RCBE^23^ was used to extract key PPG features from the recorded signals, namely amplitude, area under the curve (AUC), median upslope–downslope ratio, and median end-datum difference. Following this, statistical analysis was performed using the Kruskal–Wallis test, and feature ranking was conducted through cross-correlation and the Pearson correlation coefficient to identify those most strongly associated with arterial stiffness. For the LDF signals, the mean flux was calculated from the DC component. The following section presents and discusses these results, with emphasis on the key findings.

### Analysis of photoplethysmography signals

**Fig. 1:**
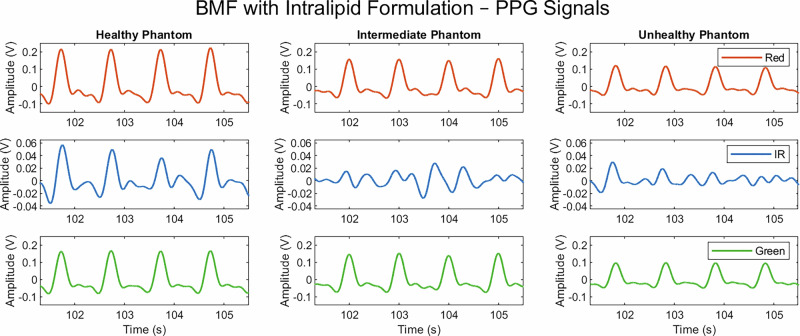
Photoplethysmography (PPG) signals acquired from healthy, intermediate, and unhealthy vessel–tissue phantoms using a multi-wavelength sensor, incorporating red, infra-red (IR), and green channels. The blood-mimicking fluid (BMF) used an intralipid formulation prepared as a 30% oil-in-water emulsion.

**Fig. 2:**
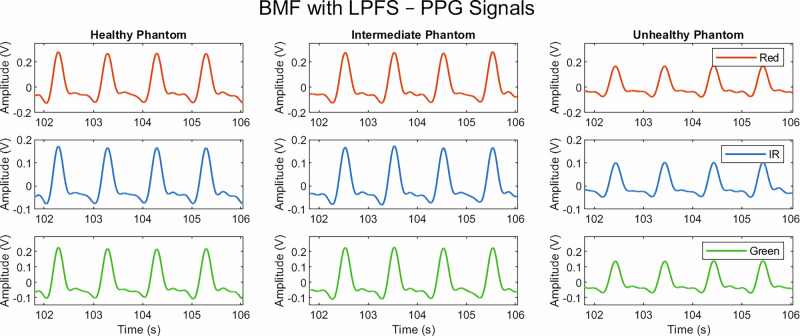
PPG signals acquired from healthy, intermediate, and unhealthy vessel–tissue phantoms using a multi-wavelength sensor, incorporating red, IR, and green channels. The BMF used LPFS at a concentration of 0.25% of the total volume.

The raw PPG signals were filtered using a second-order Chebyshev band-pass filter with a low-pass cut-off frequency of 6 Hz and a high-pass cut-off frequency of 0.5 Hz. Filtered outputs are shown in Figs. 1 and 2 for the intralipid-based and LPFS-based BMFs, respectively.

**Fig. 3:**
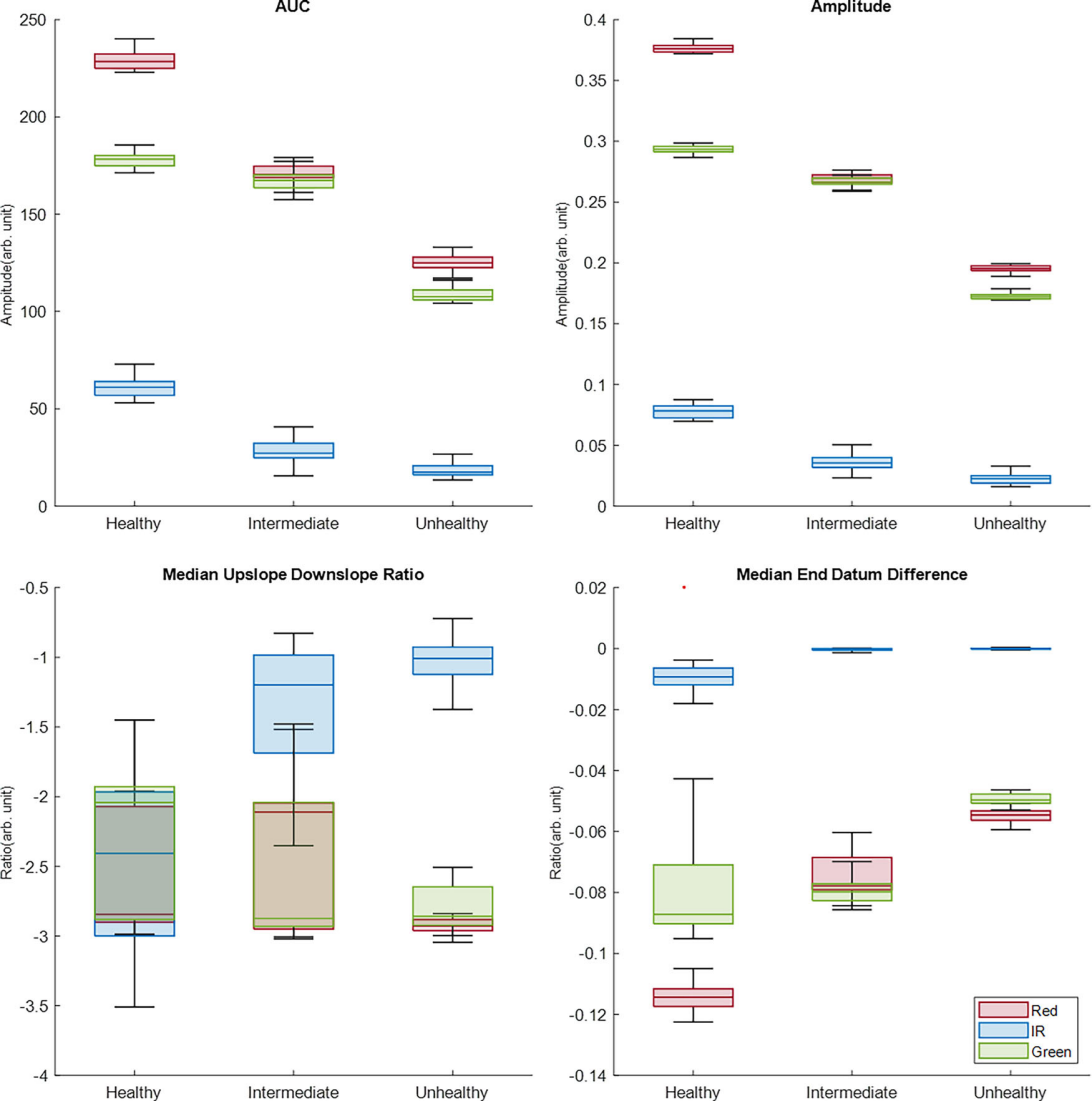
Box plot of the PPG features extracted from phantoms with the intralipid-based BMF. The red, IR, and green features are shown across phantoms arranged by decreasing elasticity: healthy (0.82 MPa), intermediate (1.48 MPa) and unhealthy (2.06 MPa).

**Fig. 4:**
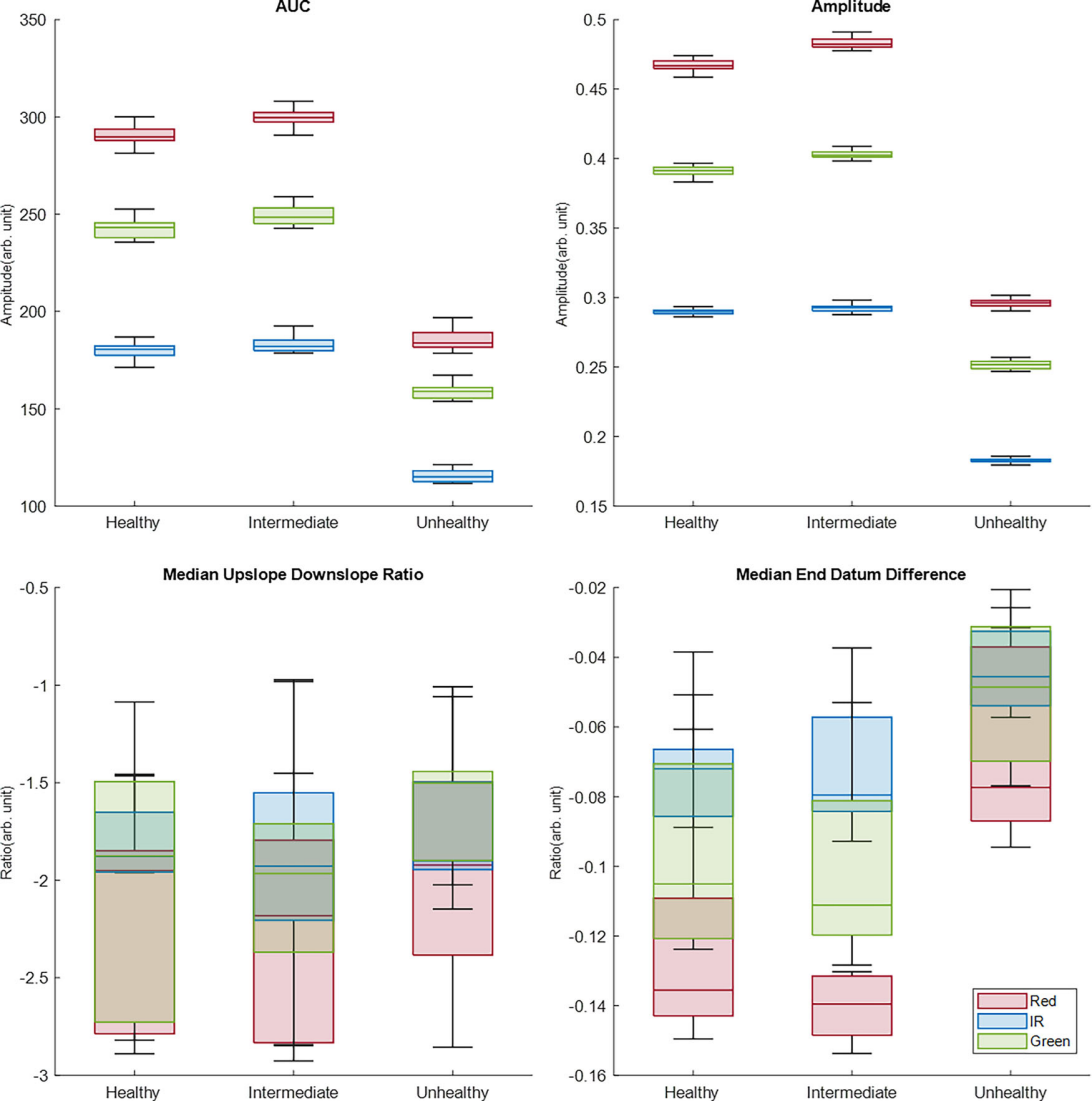
Box plot of the PPG features extracted from phantoms with the LPFS-based BMF. The red, IR, and green features are shown across phantoms arranged by decreasing elasticity: healthy (0.82 MPa), intermediate (1.48 MPa) and unhealthy (2.06 MPa).

The PPG features analysed were amplitude, AUC, median upslope–downslope ratio, and median end-datum difference. The results obtained with the intralipid-based and LPFS-based BMF are shown in Fig. 3 and in Fig. 4, respectively.

Kruskal–Wallis analysis of the extracted PPG features was performed to evaluate whether these features could distinguish between the three vessel–tissue phantoms representing different stiffness levels. The objective was to determine whether PPG could identify differences between healthy and unhealthy vascular states, as well as detect an intermediate transitional stage. Tables 1 and 2 present the Kruskal–Wallis results obtained for the intralipid-based BMF and LPFS-based BMF, respectively. As the aim of this research was to assess the feasibility of applying PPG as a non-invasive tool in primary care settings, the wavelengths were combined for simplicity. Accordingly, a single *p*-value was calculated for each feature to assess differences across stiffness levels rather than across individual wavelengths.

**Table. 1:** Kruskal–Wallis *p*-values for PPG features extracted using the intralipid-based BMF

	Healthy–intermediate	Intermediate–unhealthy	Healthy–unhealthy
AUC	*p* < 0.05	*p* < 0.05	*p* < 0.05
Amplitude	*p* < 0.05	*p* < 0.05	*p* < 0.05
Median upslope–downslope-ratio	*p* < 0.05	0.566	0.450
Median end datum difference	*p* < 0.05	*p* < 0.05	*p* < 0.05

A *p* < 0.05 was considered statistically significant. For features with *p* ≥ 0.05, the exact values are reported in the table.

**Table. 2:** Kruskal–Wallis *p*-values for PPG features extracted using the LPFS-based BMF

	Healthy–intermediate	Intermediate–unhealthy	Healthy–unhealthy
AUC	*p* < 0.05	*p* < 0.05	*p* < 0.05
Amplitude	*p* < 0.05	*p* < 0.05	*p* < 0.05
Median upslope–downslope-ratio	0.541	*p* < 0.05	0.071
Median end datum difference	0.346	0.346	*p* < 0.05

A *p* < 0.05 was considered statistically significant. For features with *p* ≥ 0.05, the exact values are reported in the table.

**Fig. 5:**
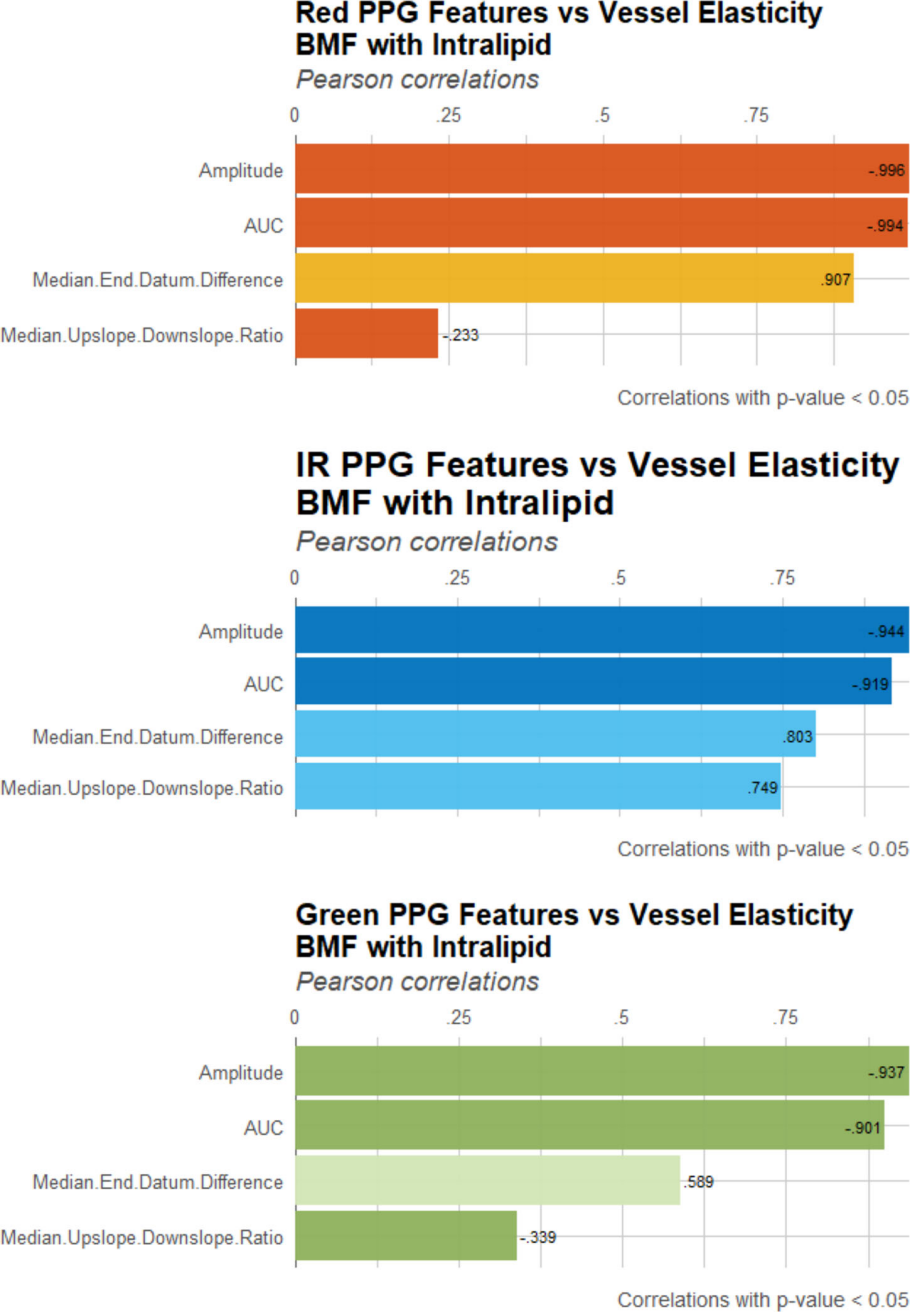
Correlation of PPG features with vessel elasticity using intralipid-based BMF. Pearson correlation coefficients (*p* < 0.05) are presented for amplitude, area under the curve (AUC), median end-datum difference, and median upslope–downslope ratio across red, IR and green wavelengths. Stronger correlations were observed in intralipid, compared to LPFS-based features, particularly for amplitude and AUC, while slope-derived features displayed weaker or inconsistent associations.

**Fig. 6:**
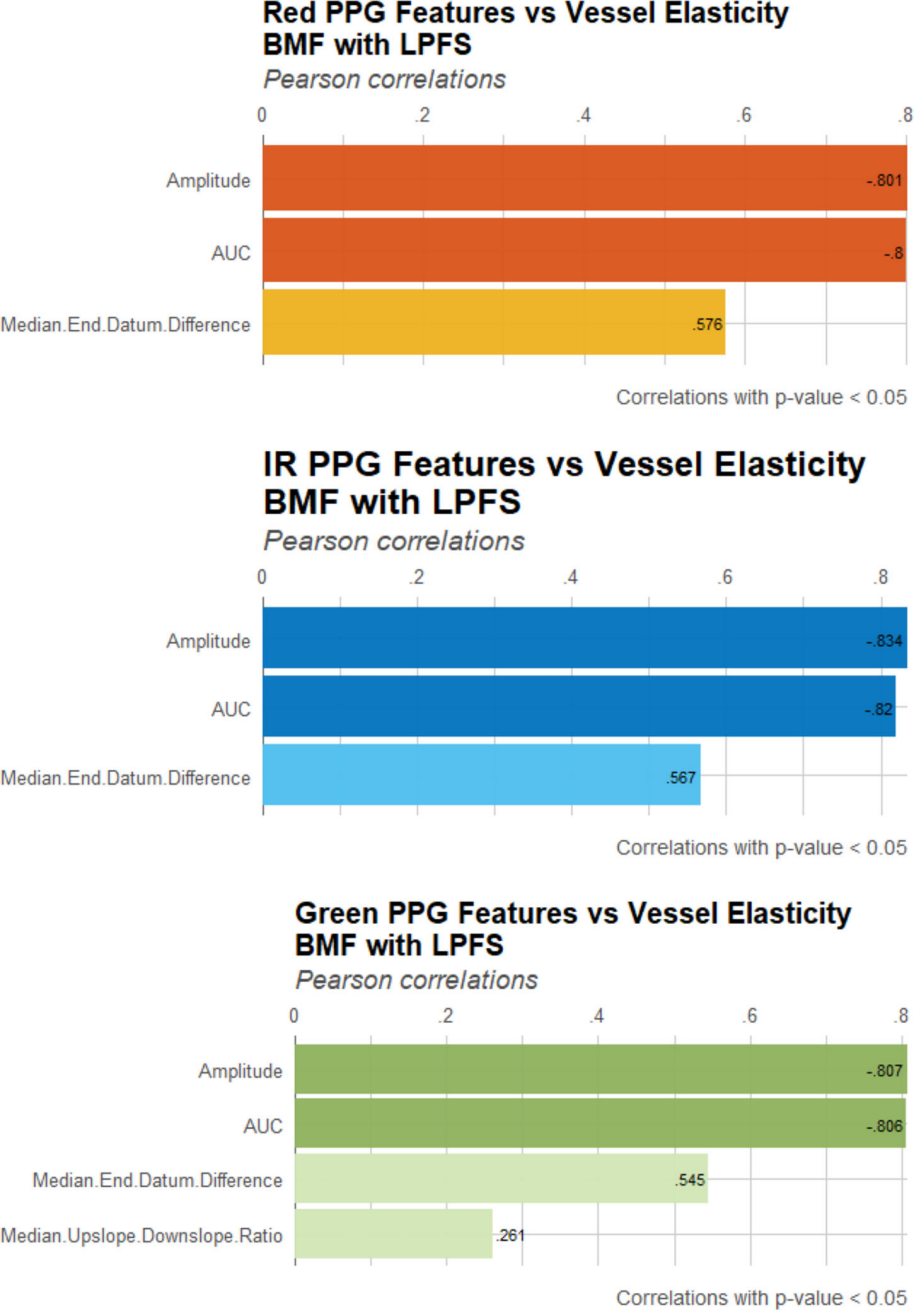
Correlation of PPG features with vessel elasticity using LPFS-based BMF. Pearson correlation coefficients (*p* < 0.05) are presented for amplitude, AUC, median end-datum difference, and median upslope–downslope ratio across red, IR and green wavelengths.

Cross-correlation analysis was performed to quantify the relationship between extracted PPG features and vessel elasticity across wavelengths and BMFs to validate the visualised box plot patterns and support the Kruskal–Wallis analysis. Features were ranked by Pearson correlation coefficients, with a significance threshold set at *p* < 0.05, presented in Fig. 5 for the intralipid-based BMF features, and Fig. 6 for the LPFS-based features. This comparison assessed whether the observed correlations were reproducible across formulations or if the type of BMF influenced the strength of association between PPG features and vessel elasticity.

### Analysis of laser Doppler flowmetry signals

**Fig. 7:**
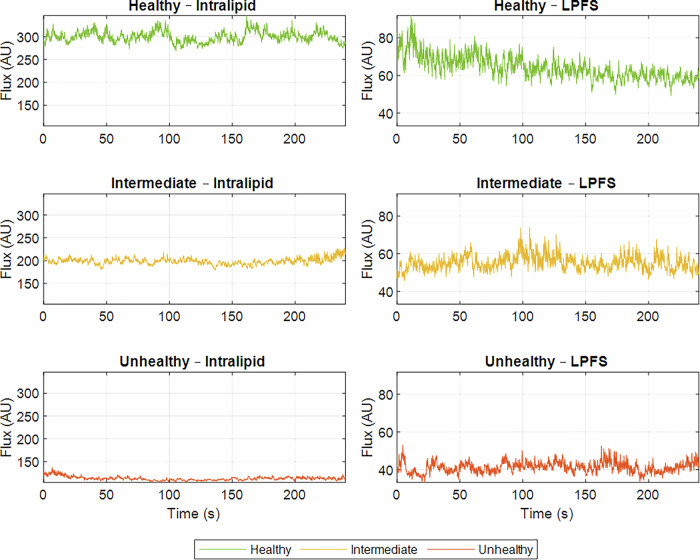
Laser Doppler flowmetry (LDF) direct current (DC) flux signals. The features are acquired from healthy (green), intermediate (yellow), and unhealthy (orange) vessel–tissue phantoms using the intralipid-based BMF (left panel) and LPFS-based BMF (right panel). The raw DC signals are displayed over the full 4-min recording.

The raw LDF signals are presented in Fig. 7 for the intralipid-based BMF and the LPFS-based BMF. The DC flux is a commonly analysed parameter in the literature^20^, ^24^ and demonstrated a relatively steady baseline across all conditions.

**Fig. 8:**
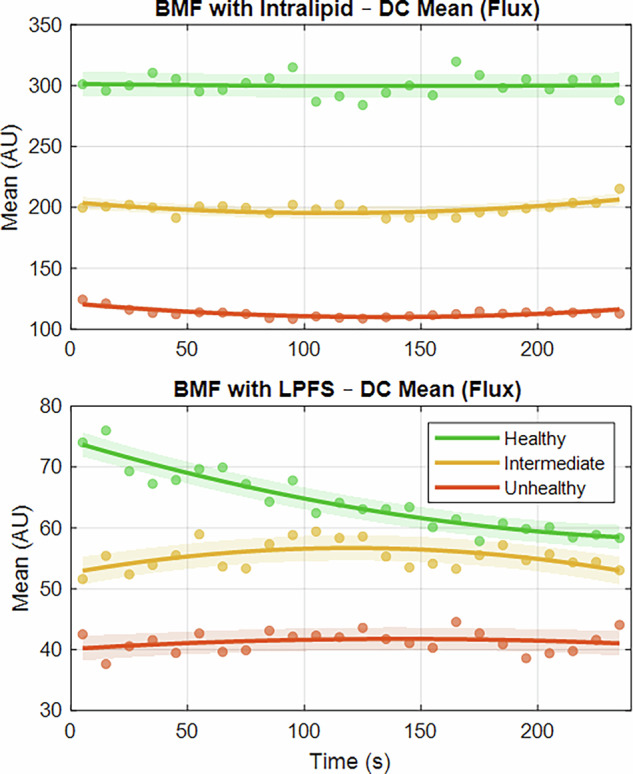
Polynomial regression fits of DC mean flux extracted from LDF signals. The features are acquired from healthy (green), intermediate (yellow), and unhealthy (orange) vessel–tissue phantoms using the intralipid-based BMF (top panel) and LPFS-based BMF (bottom panel).

Following previous literature^20^, the complementary strength of LDF alongside PPG for vascular ageing assessment was analysed by calculating and plotting DC mean flux across 10-s window averages. This is shown in Fig. 8 using a fitted polynomial per vessel–tissue phantom, for the intralipid-based and LPFS-based BMF.

Kruskal–Wallis analysis was conducted on DC mean flux to evaluate whether LDF could statistically differentiate between vessels of varying elasticities and assess its potential application in primary care. The resulting *p*-values are summarised in Table 3 for the intralipid-based BMF and the LPFS-based BMF.

**Table. 3:** Kruskal–Wallis *p*-values for LDF features extracted using the intralipid and LPFS-based BMF

DC mean flux	Healthy–intermediate	Intermediate–unhealthy	Healthy–unhealthy
Intralipid	*p* < 0.05	*p* < 0.05	*p* < 0.05
LPFS	*p* < 0.05	*p* < 0.05	*p* < 0.05

A *p* < 0.05 was considered statistically significant. For features with *p* ≥ 0.05, the exact values are reported in the table.

### Vessel classification using photoplethysmography & laser Doppler flowmetry

Multiclass classification was performed to assess whether PPG was able to predict and distinguish vessel stiffness levels, and whether combining this alongside LDF would improve predictive performance. The aim was to evaluate the potential for multimodal arterial assessment by comparing predictions based on PPG alone to results from PPG and LDF signals.

**Fig. 9:**
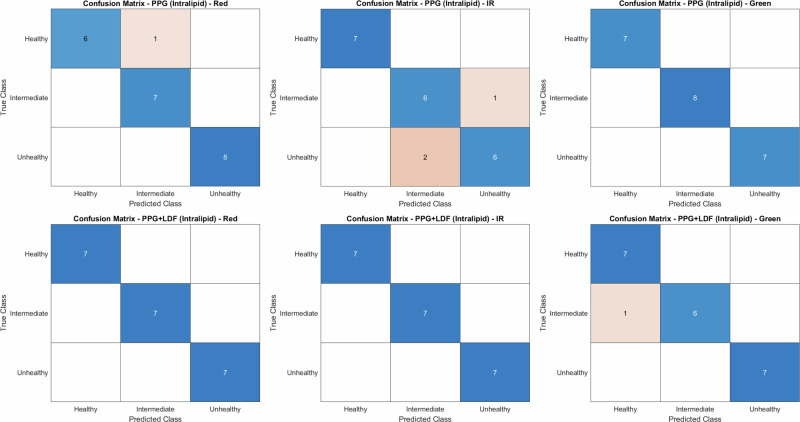
Confusion matrix for intralipid-based multiclass vessel classification. Classifications of healthy, intermediate and unhealthy phantoms are compared PPG and multimodal (PPG and LDF) algorithms.

**Fig. 10:**
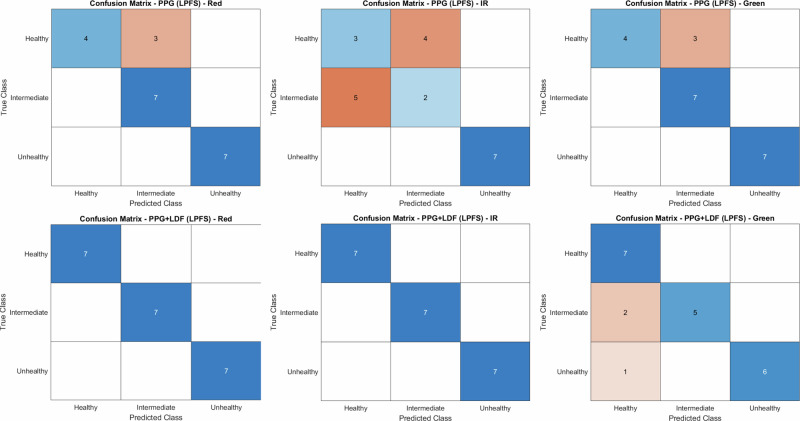
Confusion matrix for LPFS-based multiclass vessel classification. Classifications of healthy, intermediate and unhealthy phantoms are compared using PPG and multimodal (PPG and LDF) algorithms.

A linear support vector machine (SVM) was implemented within a one-vs-all error-correcting output codes (ECOC) framework. The resultant confusion matrices for intralipid and LPFS-based fluids are presented in Fig. 9 and Fig. 10, respectively.

Overall holdout accuracy and 5-fold cross-validation accuracy for the intralipid dataset are summarised in Table 4, while per-class precision, recall and F1-scores are reported in Table 5. The accuracy and metrics of the LPFS dataset are presented in Tables 6 and 7, respectively.

**Table. 4:** Classification accuracy for multiclass vessel-state prediction using the intralipid-based BMF

		Accuracy (%)	
		Holdout	5-Fold CV
Red	PPG	95.45	88
	PPG & LDF	100	91.76
IR	PPG	86.36	88
	PPG & LDF	100	100
Green	PPG	100	92
	PPG & LDF	95.24	98.61

Holdout accuracy and 5-fold cross-validation (CV) accuracy are reported for PPG–only and combined PPG and LDF feature sets across red, IR, and green wavelengths.

**Table. 5:** Per-class performance metrics for multiclass vessel-state classification using the intralipid-based BMF

		Healthy			Intermediate			Unhealthy		
		Precision	Recall	F1	Precision	Recall	F1	Precision	Recall	F1
Red	PPG	1	0.86	0.92	0.86	1	0.93	1	1	1
	PPG & LDF	1	1	1	1	1	1	1	1	1
IR	PPG	1	1	1	0.75	0.86	0.8	0.86	0.75	0.8
	PPG & LDF	1	1	1	1	1	1	1	1	1
Green	PPG	1	1	1	1	1	1	1	1	1
	PPG & LDF	0.86	1	0.93	1	0.86	0.92	1	1	1

Precision, recall and F1-score a reported for each class (healthy, intermediate and unhealthy) for PPG-only and PPG and LDF models across red, IR and green wavelengths.

**Table. 6:** Classification accuracy for multiclass vessel-state prediction using the LPFS-based BMF

		Accuracy (%)	
		Holdout	5-Fold CV
Red	PPG	85.71	81.94
	PPG & LDF	100	94.44
IR	PPG	57.14	68.06
	PPG & LDF	100	97.22
Green	PPG	85.71	86.11
	PPG & LDF	85.71	93.06

Holdout accuracy and 5-fold CV accuracy are reported for PPG–only and combined PPG and LDF feature sets across red, IR and green wavelengths.

**Table. 7:** Per-class performance metrics for multiclass vessel-state classification using the LPFS-based BMF

		Healthy			Intermediate			Unhealthy		
		Precision	Recall	F1	Precision	Recall	F1	Precision	Recall	F1
Red	PPG	1	0.57	0.73	0.7	1	0.82	1	1	1
	PPG & LDF	1	1	1	1	1	1	1	1	1
IR	PPG	0.36	0.43	0.4	0.33	0.29	0.31	1	1	1
	PPG & LDF	1	1	1	1	1	1	1	1	1
Green	PPG	1	0.57	0.73	0.7	1	0.82	1	1	1
	PPG & LDF	0.7	1	0.82	1	0.71	0.83	1	0.86	0.92

Precision, recall, and F1-score a reported for each class (healthy, intermediate and unhealthy) for PPG-only and PPG and LDF models across red, IR and green wavelengths.

## Discussion

As expected, there was a clear decrease in amplitude from the healthy to the unhealthy phantom in both BMF solutions for the PPG signals. This decrease was less pronounced in the transition from healthy to intermediate than from intermediate to unhealthy. Although the intermediate vessel was fabricated with a Young’s modulus between that of the healthy and unhealthy vessels, the corresponding change in signal amplitude between healthy and intermediate states was less evident. A notable finding was that, with the addition of intralipid to the BMF, the infra-red (IR) signal was of low quality, and the PPG waveform morphology was inconsistent across the health states. Although this may have been influenced by experimental noise, the red and green wavelengths produced distinguishable PPG signals. This may be explained by the fact that IR light penetrates deeper than red and green; however, the increased scattering induced by intralipid reduces the number of photons reaching the detector in a coherent, pulsatile pattern, thereby suppressing the IR signal. Although native blood does not contain intralipid, the scattering and refractive properties introduced by intralipid in the BMF render it a useful experimental surrogate in in vivo contexts where blood shows optical behaviour similar to intralipid-induced changes in BMF.

In contrast, the PPG IR signal did not appear to be diminished by the addition of LPFS to the BMF. Given that the volume of LPFS was comparatively lower than that of the intralipid formulation, it is likely that the intralipid itself contributed to the reduction in IR signal quality. Furthermore, when comparing the intralipid-based and LPFS-based BMFs, signals acquired with LPFS demonstrated greater amplitudes at the same wavelengths than those obtained with intralipid. This is likely due to the lower concentration of scattering agent present in the LPFS-based BMF, resulting in weaker overall scattering and allowing more photons to reach the detector. As such, reduced scattering produced larger PPG signal amplitudes.

Extracted PPG features showed a decrease in amplitude and AUC with increasing vessel stiffness across the red, IR, and green wavelengths. This reflects the reduced ability of stiffer vessels to expand, which, given that PPG is a volumetric technique, manifests as reduced amplitude and AUC. This trend is consistent with previous findings in the absence of intralipid within the BMF^19^.

However, when intralipid was introduced, the median upslope–downslope ratio did not follow the expected pattern. Although the PPG signals were morphologically consistent with expectations, the slope-derived feature appeared mathematically unstable, resulting in high variability. As this feature relies on peak detection and fiducial point identification, inaccuracies in locating these points may have contributed to variability in the slope ratios. An expected trend was observed in the red PPG signal for the median end-datum difference, which increased with stiffness, indicating a sharper signal^19^. A similar, though less pronounced, trend was observed in the green and IR signals.

When the BMF contained LPFS, the intermediate vessel–tissue phantom did not follow the expected trends for AUC and amplitude, where a progressive decrease was expected with increasing stiffness. Although a reduction was observed from the healthy, elastic vessels to the stiff, unhealthy vessels, the intermediate vessel response was closer to the healthy case and, in some instances, slightly elevated, as shown in Fig. 4. This behaviour has also been reported previously^19^, where the red, IR and green wavelengths showed similar values between the healthy and intermediate levels for AUC, and in terms of amplitude, the red signal increased from the healthy to intermediate state.

In contrast to the intralipid-based BMF, both the median upslope–downslope ratio and the median end-datum difference demonstrated increased variability across all vessel states and wavelengths. The upslope–downslope ratio is particularly sensitive to the downslope region of the PPG waveform, where the signal is more susceptible to noise and baseline drift, which can complicate accurate pulse segmentation. The introduction of LPFS increases scattering and may introduce inaccuracies in fiducial point identification, thereby affecting downslope estimation. This instability likely explains the absence of consistent trends across the stiffness levels. Similarly, the end datum difference displayed greater variability in the LPFS-based BMF compared with the intralipid-based BMF. This was most evident in the healthy and intermediate states, although the unhealthy phantom also showed increased variability. These findings suggest that while the end-datum difference may retain some discriminatory value between vessel states^19^, its reliability diminishes when additional scattering agents are introduced to the BMF.

Using the intralipid-based BMF, statistically significant differences were observed between the healthy and intermediate vessel–tissue phantoms across all extracted PPG features (as shown in Table 1). In contrast, previous work in which the BMF did not include intralipids reported no significant differences between the healthy and intermediate phantoms for AUC and amplitude^19^. Furthermore, while earlier results showed that all features yielded *p* < 0.05 when comparing the intermediate and unhealthy phantoms, as well as the healthy and unhealthy^19^, the intralipid-based BMF did not demonstrate a significant difference for the median upslope–downslope ratio. This discrepancy may reflect the instability of this feature, as illustrated in Fig. 3, whereby high variability was observed.

Interestingly, although the LPFS-based BMF produced higher PPG signal amplitudes, the intralipid-based BMF showed a greater number of statistically significant PPG features. The median upslope–downslope ratio did not demonstrate significant differences between either the healthy and intermediate or the healthy and unhealthy phantoms, highlighting the need to explore additional features. Furthermore, although the median end-datum difference identified a significant difference between the healthy and unhealthy vessel–tissue phantoms, the intermediate stage did not differ significantly from either extreme. Therefore, while the median end-datum difference was able to discriminate between the two extremes of elasticity, additional features may be required to capture transitional stages if the LPFS-based BMF is to be used in future investigations.

Comparing the two BMF formulations, intralipid consistently produced stronger correlations across all wavelengths, particularly amplitude and AUC. The LPFS formulation also demonstrated significant correlations; however, their overall strength was comparatively weaker. This suggests that intralipid functions as a more reliable optical medium for accentuating the sensitivity of PPG features to vascular elasticity.

Amplitude and AUC emerged as the most reliable indicators of vessel stiffness, exhibiting strong negative correlations consistently across wavelengths and both formulations, supporting the Kruskal–Wallis test and box plot distributions. The end-datum difference also showed a consistent positive correlation, though its magnitude fluctuated between wavelengths and BMFs. The median upslope–downslope ratio was the least consistent feature, with varying correlation directions and, in some cases, not reaching statistical significance. This highlights the susceptibility of slope-derived features to noise and errors in fiducial point detection, which limits their utility as stiffness markers compared to those based on amplitude and area.

The cross-correlation results align with the findings from both the Kruskal–Wallis test and the box plot distributions, indicating that intralipid-based BMF provides stronger statistical validation for arterial stiffness detection using PPG-derived features. Furthermore, amplitude and AUC were consistently shown to be more reliable than datum and slope features. These PPG features could potentially be investigated alongside LDF signals to identify similar or contrasting physiological patterns.

For both formulations, the DC amplitude of the LDF signals decreased progressively from the healthy to the unhealthy states. Previous studies have similarly reported that perfusion levels decline from healthy individuals to those with vascular pathologies^24^. Notably, the intralipid-based BMF consistently produced higher flux values compared to the LPFS-based BMF under equivalent vessel–phantom conditions. Moreover, the intralipid formulation demonstrated a more pronounced reduction in flux across the healthy, intermediate, and unhealthy states, suggesting improved differentiation between vascular conditions. Such clearer separation may be advantageous in primary care settings, where larger signal variations could facilitate clinical interpretation. This difference may, however, be attributed to the higher concentration of the scattering agent present in the intralipid-based BMF relative to the LPFS-based formulation.

The LDF DC mean flux yielded promising results, with clear differences between each health stage of the vessel–tissue phantoms, consistent with the statistically significant differences observed (*p* < 0.05). As vascular health declined, flux levels decreased, consistent with the hypothesis that stiffer vessels have lower flow rates due to increased resistance, as explained by Poiseuille’s law^14^. Accordingly, the DC mean flux from the vessel–tissue phantoms reflected this pattern in both the intralipid- and LPFS-based BMFs. The intralipid-based BMF showed a greater range of flux variation between stiffness levels, likely due to the higher concentration of intralipid used.

Classification of the vessel states using PPG alone with both intralipid and LPFS-based fluid showed that the red and green channels provided the strongest discriminatory performance, particularly for the intermediate and unhealthy phantoms. Under intralipid, this was reflected by high holdout accuracies for red (95.45%) and green (100%), showing only occasional healthy states misclassified as intermediate. Under LPFS, red and green remained the strongest PPG channels (both 85.71% holdout). In contrast, the IR channel showed the weakest separation, with a holdout accuracy of 57.14% in LPFS and 86.26% in intralipid.

When LDF-derived features were combined with the red and IR PPG feature sets, classification performance improved, most notably for IR. For both intralipid and LPFS datasets, adding LDF yielded classification metrics of 1 for precision, recall and F1-scores, for the red and IR models, indicating zero false-positives and false-negatives. Overall, intralipid-based measurements yielded higher baseline classification accuracy than LPFS; however, improvements were evident for both formulations when LDF features were incorporated, supporting the added value of a multimodal approach.

According to Poiseuille’s law (Eq 1), aged vessels, which are less compliant with increased resistance, should demonstrate lower flow rates in comparison to young, healthy elastic vessels^14^. Consistent with this, LDF measurements in both the intralipid and LPFS setups showed that greater vessel stiffness was associated with lower flux. To substantiate this mechanistic relationship, future studies should validate these findings alongside direct pressure measurements. According to the multi-class classification results, LDF may be integrated with PPG during in vitro investigations using vascular phantoms to support the assessment and prediction of vascular health states. This study provides a foundation for researchers seeking to compare multimodal PPG and LDF approaches for assessing vascular ageing.

Optimising the scattering properties of the BMF is critical to achieving a formulation suitable for both modalities. In this study, two additives were investigated: intralipid and LPFS. Both formulations produced LDF signals that varied with vessel stiffness, with mean flux levels decreasing as stiffness increased. Although this trend was observed in both BMFs, the intralipid-based BMF demonstrated stronger separation between vessel states and a wider range of flux values. A negative effect of intralipid was observed in the PPG signals, characterised by reduced amplitude across all wavelengths. This effect was most pronounced in the IR signals, where visual degradation of pulsatility was also evident. In contrast, these effects were not present when using the LPFS formulation. Despite the suppressed IR signal when using intralipid-based BMF, extracted features had stronger correlations with vessel stiffness than those produced with LPFS-based BMF. To optimise signal quality across both techniques, future studies should investigate varying intralipid concentrations. The observed differences may also be related to the lower volume of LPFS used in this study compared to the higher concentration of intralipid within the BMFs. As such, future work should investigate a range of concentrations for both scattering agents. Although large pulsatile vessels are predominantly located within the dermis and subcutaneous tissue^25^, vessel depth remains an important consideration for optical measurements. For PPG, increasing vessel depth reduces the fraction of photons that penetrate to the vessel and return to the photodetector, due to increased optical path length and attenuation within the overlying tissue. Similarly, for LDF, where the detected signal depends on the effective measurement depth^16^, greater vessel depth is expected to reduce the signal quality, thereby making it more challenging to discriminate between different vessel stiffness levels.

Furthermore, it should be noted that, in clinical use, LDF predominantly reflects perfusion within the cutaneous microcirculation. In the present study, low-pass filtering was applied to the LDF signals to attenuate high-frequency components potentially introduced by pump actuation or mechanical vibration, thereby reducing non-physiological harmonic content that would not be expected during native cardiac contraction. Nevertheless, an important limitation is that, under the current phantom geometry, the LDF measurements likely sampled a signal dominated by the vessel region rather than cutaneous perfusion as in vivo. Consequently, the observed LDF–stiffness associations should be interpreted within the context of this phantom configuration and require validation in clinical studies.

These findings support the integration of LDF with PPG for vascular assessment, enabling more accurate prediction of vascular health states. However, limitations remain, including the signal processing and motion artefact challenges associated with LDF^9^, as well as potential cross-interference between sensors, which may be minimised by multiplexing. Synergising the strengths of both modalities may improve diagnostic accuracy. Although certain features across stiffness levels were not statistically distinguishable, and overlap was observed for the intermediate state, integrating the two modalities could enable a more accurate assessment of vascular health if translated to GP settings, as indicated by the multi-class classification. In instances where findings diverge between modalities, GPs could use this conflict as a trigger for referral to more comprehensive vascular assessment, which is typically undertaken in hospital settings^9^. Future work should explore combining both modalities into a single device to minimise instrumentation and time and ensure practicality in primary care settings.

This study marks an early step toward integrating LDF with PPG for the assessment of vascular ageing, with the aim of developing a multimodal approach suitable for primary care settings. Two scattering agents were incorporated into a BMF to evaluate both modalities: intralipid- and LPFS-based formulations. Although the intralipid-based BMF suppressed the IR component of the PPG signal, it produced larger reductions in DC flux between vascular health states compared to the LPFS-based BMF. The differences in flux values between healthy and diseased conditions may enable clearer clinical interpretation in primary care settings.

Future work should explore optimising scattering concentrations, either by reducing intralipid levels to minimise IR suppression or by increasing LPFS concentrations, as the present study was constrained by using lower LPFS levels than intralipid. Additional experiments at lower flow rates are also recommended; while PPG signals may be compromised under such conditions, LDF may maintain performance and reduce the risk of saturation. Furthermore, the in vitro system should be modified to enable assessment of microcirculation, as the present study obtained flux measurements from macro-scale vessels only. Applying frequency-domain and wavelet analyses could enhance the characterisation of LDF signals in future studies. To evaluate the feasibility of LDF and PPG, individually or in combination, for point-of-care application, in vivo studies are recommended, commencing with healthy participants and progressing to patients with established vascular risk.

It is acknowledged that the BMFs were not optically characterised in this study. The measurements were conducted with the primary objective of investigating disease progression associated with mechanical alterations, predominantly arterial stiffening, rather than assessing the influence of BMF optical properties. For each experiment, the BMF composition was kept constant while the mechanical properties of the phantom were varied. It is suggested that future work should formulate BMFs that are both optically and mechanically characterised to better approximate in vivo haemodynamic. Future work should also include optical characterisation of the vessel–tissue phantoms. In the present study, while the optical properties of the vessel–tissue phantoms were not quantified, mechanical characterisation was performed to capture mechanical changes relevant to vascular ageing. However, future work would benefit from vessel–tissue phantoms that more realistically replicate human physiology, including characterised optical properties. In addition, the phantoms could be refined to incorporate distinct intima, media and adventitia layers, allowing closer replication of human arterial structure.

This work provides a foundation for the integration of LDF, either independently or in combination with PPG, as a potential tool for the non-invasive assessment of vascular health.

## Methods

Custom vessel–tissue phantoms, replicating the mechanical properties of the human femoral artery, were incorporated into an in vitro cardiovascular model simulating the lower body circulation. PPG and LDF signals were recorded from the system for each phantom. The acquired signals were examined and analysed to evaluate their statistical significance.

### Custom femoral artery phantoms

**Fig. 11:**
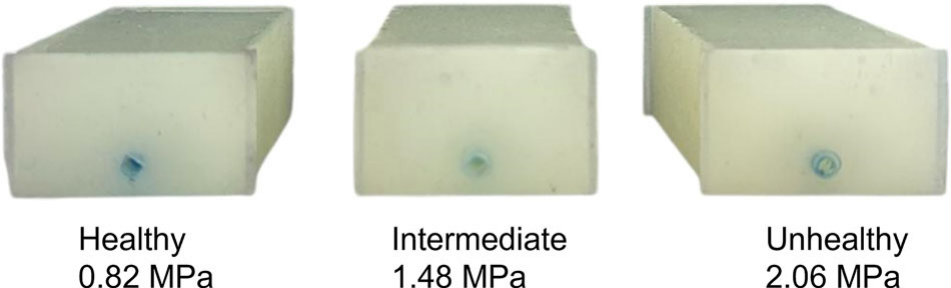
Fabricated vessel–tissue phantoms. The phantom on the left represents a healthy femoral artery, with a vessel characterised by a Young’s modulus of 0.82 MPa. The central phantom corresponds to an intermediate pathological stage, incorporating a vessel with a Young’s modulus of 1.48 MPa. The phantom on the right represents an atherosclerotic femoral artery, with a vessel modelled to have a Young’s modulus of 2.06 MPa.

Three preconstructed phantoms, as shown in Fig. 11, developed by the RCBE^1^, ^19^, were used in this study due to their mechanical resemblance to human femoral arteries. These vessel–tissue phantoms featured embedded vessels with inner and outer diameters of 2.95 and 3.96 mm, respectively, consistent with values reported in the literature, where outer diameters range from 3.9 to 8.9 mm^1^, ^26^. The surrounding tissue was consistent across all phantoms; however, the embedded vessels, located at a depth of 3 mm within the phantom, differed in mechanical stiffness, as characterised by their respective Young’s modulus. One vessel, with a Young’s modulus of 0.82 MPa, was selected to represent a healthy femoral artery, while another, with a modulus of 2.06 MPa, was used to model an unhealthy atherosclerotic artery. A third vessel, representing an intermediate pathological state, was fabricated with a measured Young’s modulus of 1.48 MPa. These values were obtained using a Universal Testing System (Instron 5944, Norwood, MA, USA) in a tensile test configuration, following the ASTM D412 standard^27^. All phantoms were fabricated in a controlled laboratory setting and were designed to replicate physiological conditions as closely as possible, based on existing literature^28^. To minimise batch variability, all phantoms were fabricated using the same base elastomer batch; vessel stiffness was tuned solely by varying the additive (hardener) ratio for the embedded vessels, while the surrounding tissue material was kept constant.

### Setup of the in vitro cardiovascular system

The in vitro cardiovascular system was developed based on previously described methods^19^, ^29^, with the integration of a LDF device (moorVMS-LDF, Moor Instruments Ltd, Devon, UK). The vascular model was designed to replicate the aorta branching into the femoral and tibial arteries, thereby mimicking the lower limb vasculature, where PAD most commonly occurs^30^. A pulsatile pump (PD-1100, BDC Laboratories, Wheat Ridge, CO, USA) operating at 60 bpm was connected to simulate physiological pulsatile flow.

### Blood-mimicking fluid

Two formulations of BMF were evaluated to determine which offered superior performance in in vitro PPG and LDF experiments. Both BMFs were prepared at the RCBE using a previously established protocol^19^, with the addition of a scattering agent. Each formulation contained Wright stain (Thermo Fisher Scientific Inc., Waltham, MA, USA), Congo red powder (BDH Chemicals Ltd., Poole, UK), and Indian ink (Jackson’s Art Supplies, London, UK), with deionised water serving as the base solvent. The scattering medium was either LPFS (Moor Instruments Ltd, Devon, UK) or a laboratory-prepared intralipid, produced via oil-in-water emulsion with lecithin (Product Code: 10494501, Thermo Fisher Scientific Inc, Waltham, MA, USA).

The LPFS contains the non-ionic surfactant Tween 20 (Sigma-Aldrich, St. Louis, MO, USA) and OptiBind™ polystyrene microspheres (Seradyn Inc., Indianapolis, IN, USA) that undergo Brownian motion, thereby enhancing optical scattering. This formulation was incorporated at a concentration of 0.25% of the total BMF volume. The intralipid formulation incorporated a 30% oil-in-water emulsion, which was prepared in the laboratory following a previously described method and selected as an intermediate concentration within the range reported in the literature^31^. To minimise the risk of phase separation, experiments involving intralipid were completed within a short period following BMF preparation. To maintain homogeneity throughout the protocol, the intralipid was gently mixed prior to acquisition.

### Sensor configuration

**Fig. 12:**
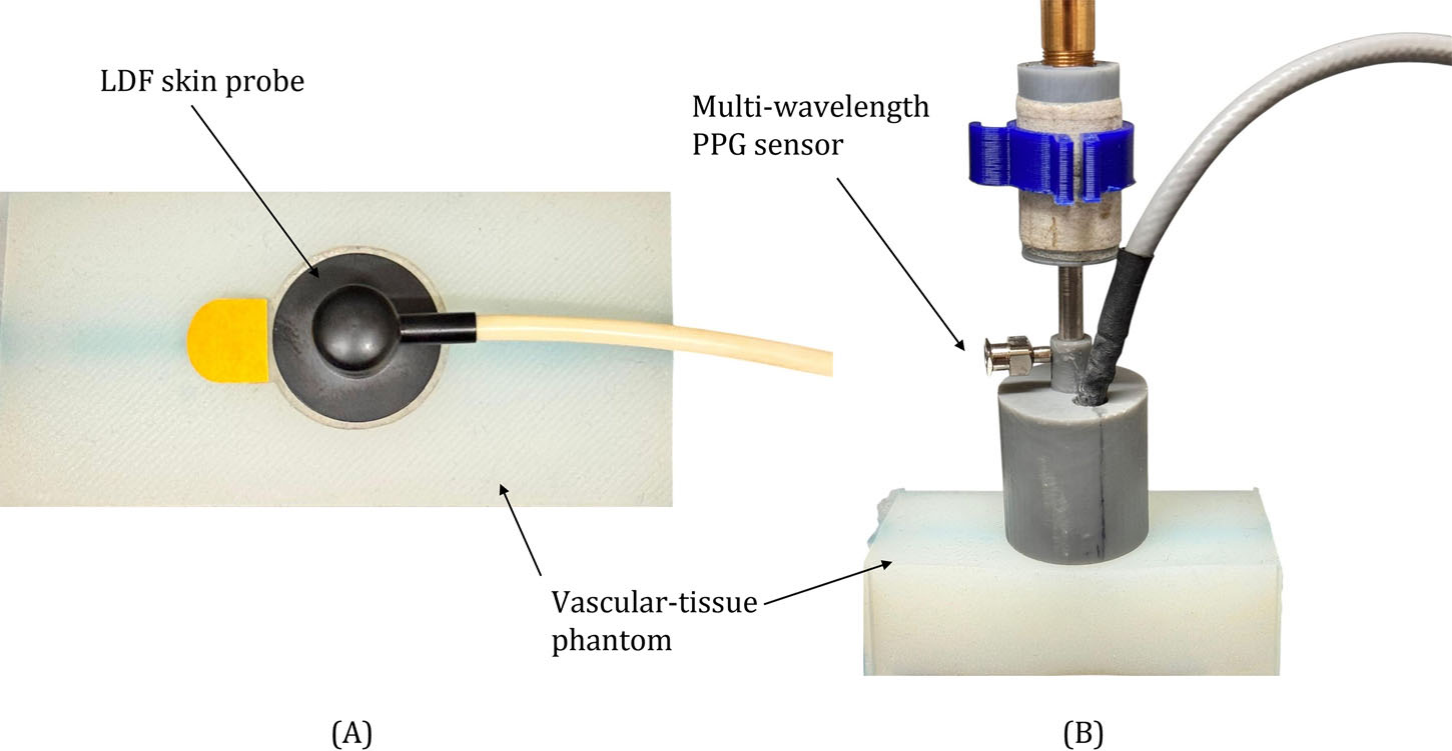
PPG and LDF sensor placement. Configuration of the LDF (**A**) and PPG (**B**) sensors on the vessel–tissue phantom. The PPG sensor and LDF probe were alternated to obtain sequential readings and prevent optical interference.

The LDF and PPG sensors were positioned on the custom-made phantoms, as illustrated in Fig. 12. The PPG sensor and LDF probe were alternated to obtain readings and prevent any optical interference between the light sources of the two devices. To avoid cross-interference, data collection was performed sequentially, with each recording lasting 4 min. The PPG sensor was connected to acquire signals at three wavelengths: green (530 nm), red (655 nm), and IR (940 nm). It was positioned above the phantom and interfaced with a PPG acquisition system operating at a sampling frequency of 2000 Hz^32^. The LEDs, which determined the light intensity of the sensor, were set to 30 mA. The LDF system was connected in single-channel mode, and a skin probe was secured to the phantom. Tissue perfusion (Flux) data were collected from the macro-scale vessel using a laser diode operating at 785 nm, with a maximum output power of 1.3 mW. The Flux smoothing time constant was set to 1.0 s^33^.

### Feature extraction

Building on prior work^19^, four primary PPG features were selected for analysis in the present study: amplitude, AUC, median upslope–downslope ratio, and median end-datum difference. These features were originally identified and applied for their relevance to vascular ageing assessment^19^, and have been re-examined in this study to extend that investigation. The rationale for selecting these specific features was to evaluate whether they alone provide sufficient sensitivity for detecting vascular ageing, or whether additional parameters may be required. As previously reported, ageing vessels are associated with a reduction in amplitude, AUC, and the median upslope–downslope ratio, as well as an increase in the end-datum difference when measured using PPG^19^.

For the LDF signal, and in accordance with the literature^20^, the direct current (DC) component was analysed to yield a mean flux by dividing the signal into 10-s windows. This was modelled using degree-2 polynomial regression. It was expected that, as vessels stiffen and vascular resistance increases, flow rate would decrease. The reduced flow rate was hypothesised to present a reduction in flux levels when transitioning from healthy to unhealthy vessel–tissue phantoms.

### Statistical analysis and vessel classification

The resultant features were statistically analysed to assess whether they could discriminate between phantoms with differing vascular health statuses. Features obtained from each device were analysed using the Kruskal–Wallis test, and the PPG features were further subjected to cross-correlation analysis. These methods were chosen to assess statistically significant differences across health states for both modalities and to identify the most significant features. Demonstrating such differences in both devices would support their combined use as complementary diagnostic tools in GP settings, where dual confirmation could enhance the reliability of vascular ageing assessments and potentially aid in identifying intermediate stages of vascular decline.

The Kruskal–Wallis test is a nonparametric method for one-way analysis of variance used to assess whether statistically significant differences exist among more than two independent groups. A *p*-value less than 0.05 was considered statistically significant^34^. Cross-correlation analysis was conducted to evaluate the relationship between each PPG feature and arterial stiffness, which were ranked according to their significance^29^. The strength and direction of these relationships were quantified using Pearson correlation coefficients^35^.

In addition to a feature-level statistical testing, a supervised vessel-prediction approach was implemented to evaluate whether feature sets could be used to classify vessel-state directly. Multiclass classification was performed with the three vessel–tissue phantoms using windowed feature vectors extracted from each recording. Models were trained separately for each PPG wavelength to avoid crossover between wavelength-dependent feature distributions. Two input configurations were evaluated: PPG-only and PPG combined with LDF, where the LDF mean flux was included with the corresponding PPG feature set. Predictive performance was evaluated using a random 70/30 holdout split to generate confusion matrices and overall accuracy, with 5-fold cross-validation used to provide an additional estimate of performance stability. In addition to overall accuracy, class-wise precision, recall and F1-score were computed.

## Data Availability

The datasets generated during and/or analysed during the current study are available from the corresponding author on reasonable request.

## Code availability

The underlying code for this study is not publicly available but may be made available to qualified researchers on reasonable request from the corresponding author.
